# Brain deposition of gadobutrol in children—a cross-sectional and longitudinal MRI T1 mapping study

**DOI:** 10.1007/s00330-022-09297-y

**Published:** 2022-12-15

**Authors:** Daniel Gräfe, Stefan-Horia Simion, Maciej Rosolowski, Andreas Merkenschlager, Jens Frahm, Dirk Voit, Franz Wolfgang Hirsch

**Affiliations:** 1 Department of Pediatric Radiology, University Hospital, Leipzig University, Liebigstraße 20a, 04103 Leipzig, Germany; 2 Department of Radiology, Municipal Hospital, Dessau, Germany; 3 Institute for Medical Informatics, Statistics and Epidemiology, Leipzig University, Leipzig, Germany; 4 Department of Neuropediatrics, University Hospital, Leipzig, Germany; 5 Biomedizinische NMR, Max-Planck-Institut für Multidisziplinäre Naturwissenschaften, Göttingen, Germany

**Keywords:** Gadolinium, Brain, Children, Magnetic resonance imaging

## Abstract

**Objectives:**

Depositions of linear gadolinium-based MRI contrast agents are readily visible in T1-weighted MRIs of certain brain regions in both adults and children. Macrocyclic contrast agents such as gadobutrol have so far escaped detection by qualitative MRI in children. This study aimed to assess whether there is evidence for deposition of gadobutrol in children using quantitative T1 mapping.

**Methods:**

This retrospective study included patients, naive to other gadolinium-based contrast agents than gadobutrol, who had received gadobutrol as part of a clinically indicated MRI. For each patient, T1 relaxation times at 3 T were measured using single-shot T1 mapping at two time points. In each of six brain regions, age-adjusted T1 relaxation times were correlated with a number of previous gadobutrol administrations. To combine interindividual, cross-sectional effects with intraindividual, longitudinal effects, both linear mixed model and generalized additive mixed model were applied.

**Results:**

One hundred four examinations of 52 children (age median 11.4, IQR 6.3–15, 26 female) with a median of 7 doses of gadobutrol in the history of their neurological or neurooncological disease were included. After correction for age and indeterminate disease-related effects to T1 time, a negative correlation of T1 time with the number of gadobutrol doses administered was observed in both mixed models in the putamen (beta − 1.65, *p* = .03) and globus pallidus (beta − 1.98, *p* = .012)

**Conclusions:**

The results indicate that in children, gadobutrol is deposited in the globus pallidus and putamen.

**Key Points:**

*• Previous gadobutrol administration correlates with reduced T1 relaxation times in the globus pallidus and putamen in children.*

*• This decreased T1 might be caused by gadobutrol retention within these gray-matter nuclei.*

**Supplementary Information:**

The online version contains supplementary material available at 10.1007/s00330-022-09297-y.

## Introduction

In MRI, gadolinium-based agents reduce the proton T1 relaxation times of affected tissue. They are considered safe when used as MRI contrast agents [1, 2]. Kanda et al’s 2014 demonstration of hyperintensities in the globus pallidus (GP) and nucleus dentatus (ND) in native T1-weighted images of patients after multiple administrations of gadolinium-based contrast agents was a cause of concern in health care professionals and the public [3]. The presumed cause of the hyperintensities was a deposit of gadolinium, which has been confirmed in several retrospective MRI, histopathologic, and animal studies [4–7].

Gadolinium-based contrast agents can be grouped into macrocyclic and linear, with the former demonstrating higher thermodynamic and kinetic stability due to their cubic structure [8]. Gadolinium deposits mainly occur in gray-matter nuclei in adults and children, with significantly stronger effects of linear chelate complexes than macrocyclic complexes in adults [5, 7, 9]. Therefore, after corresponding warnings by the FDA in 2017, linear gadolinium-containing contrast media are almost exclusively replaced by macrocyclic contrast media such as gadobutrol, where deposits are much more difficult to detect [10–14].

Since histopathologic findings of gadolinium deposition in the brain are only posthumous, they are loaded with confounding factors and difficult to collect. Therefore, in vivo morphologic correlates for possible deposition are consulted. A simple intensity analysis based on T1-weighted sequences, which are predominantly used in MRI studies with a contrast agent, relies on proportions of an index tissue to a reference tissue, as respective intensities only represent relative values. This approach is prone to false-positive as well as false-negative results [15]. Using this qualitative technique, evidence of gadobutrol deposition could be found neither in adults nor children (9–12). In contrast, the absolute measurement of T1 relaxation times—T1 mapping—allows for a quantitative assessment of the gadolinium effect on T1 and therefore promises higher sensitivity. In adults, T1 mapping demonstrated changes in T1 relaxation time after multiple gadolinium administrations, including gadobutrol [16, 17]. However, no T1 mapping study has yet been reported for children whose developing brains may have different deposition patterns. One limiting factor for those studies is that the infant brain in the first years of life already undergoes a physiological T1 time decline mostly as a result of the emerging myelination [18]. As a result, the fact of gadobutrol deposition in children’s brain tissue is still controversial. Besides T1 mapping techniques such as MP2RAGE [19] or synthetic MRI [20], a rapid and highly accurate T1 mapping technique termed T1FLASH (T1 fast low-angle shot) has been recently described [21].

The aim of the study was to identify changes in T1 relaxation time as indicators of gadobutrol deposition in the brain of children, using a rapid and highly accurate T1 mapping technique for which pediatric normative values have been published [22].

## Materials and methods

### Cohort

The retrospective study covered a period from September 2019 to January 2022 at a pediatric tertiary center. The study was approved by the local ethics committee (367/19-ek).

The participants comprised children and adolescents up to 18 years of age who had obtained two cerebral T1 MRI examinations with at least one gadobutrol administration between these scans. Patients with very extensive pathology, in which more than three regions of interest (ROIs) had to be discarded due to pathologic alterations, were excluded. Patients with administration of a contrast agent other than gadobutrol (Gadovist; Bayer Schering Pharma) in their history were also excluded. An interval between gadobutrol administration and T1 mapping of less than 7 days was another exclusion criterion, to ensure complete primary renal excretion [23]. Furthermore, patients with status post cranial radiotherapy were excluded, as radiation-induced injury may alter T1 [24]. Moreover, since gadobutrol is renally eliminated, patients with renal insufficiency (estimated glomerular filtration rate below 90 mL/min/1.73 m^2^) were excluded.

**Fig. 1:**
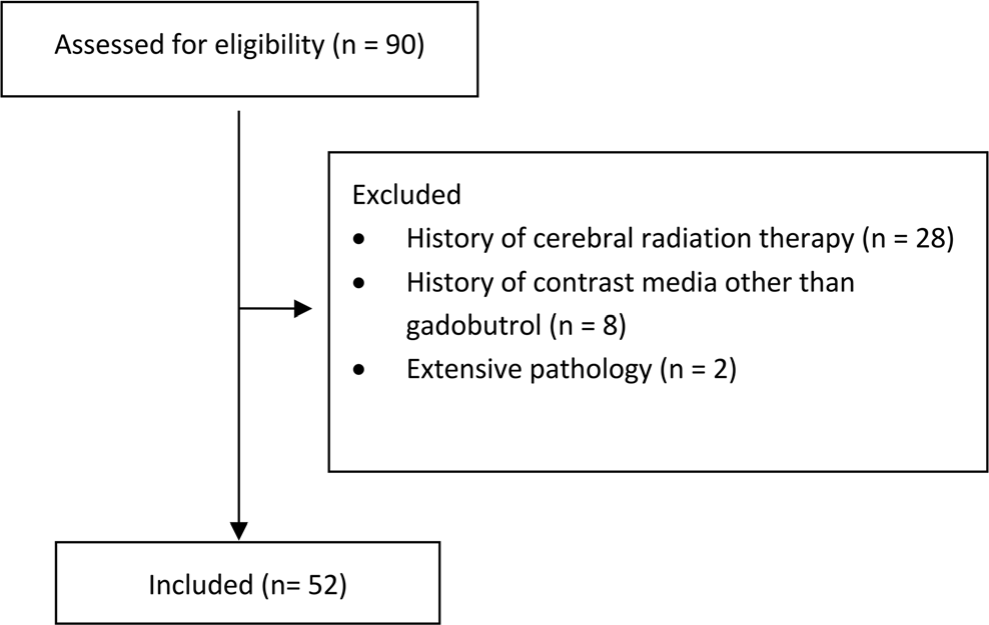
Flowchart for patient inclusion

A total of 90 patients received at least two examinations with T1 mapping during the study period. Twenty-eight patients were excluded because of a history of radiotherapy, eight due to prior administration of another contrast agent and two because of extensive pathology (Fig. 1). No patient was excluded due to renal insufficiency. Thus, 52 patients (median age 11 years, IQR 6–15, 26 female) were included in the study.

In order to generate age-adjusted T1 values, we employed a normative population already studied using the same methodology [22]. The latter cohort consisted of 100 patients with a median age of 11 years (IQR 7–15, 54 female).

### MRI

All examinations were performed at 3 T (Prisma fit, Siemens Healthcare) with a 64-channel head coil. T1 mapping with T1FLASH employs a single slice-selective 180° inversion pulse and probes the resulting inversion recovery process by a continuous series of spoiled fast low-angle shot (FLASH) images with randomized radiofrequency phases [21, 25]. A highly undersampled radial golden angle trajectory (TR 3.51 ms, TE 2.24 ms, voxel size 1 × 1 × 3 mm, flip angle 6°, 17 radial spokes) yielded an individual image acquisition time of 60 ms. The estimation of serial images by regularized nonlinear inversion [26] was followed by denoising and pixelwise fitting. Online reconstruction, visualization, and storage of T1 maps without the need for any user interference were ensured by a dedicated GPU computer bypassing the host of the MRI system. T1 mapping of the whole brain required approximately 2.5 min.

Conventional sequences included a T1 Magnetization Prepared Rapid Acquisition with Gradient Echoes (T1 MPRAGE) sequence, T2 turbo spin-echo (TSE) sequence with and without a fluid attenuation inversion recovery (FLAIR), and an echoplanar diffusion weighted imaging sequence (EPI-DWI). Weight-adapted (1 mmol/kg body weight) gadobutrol was administered solely as clinically necessary.

### T1 map analysis

**Fig. 2:**
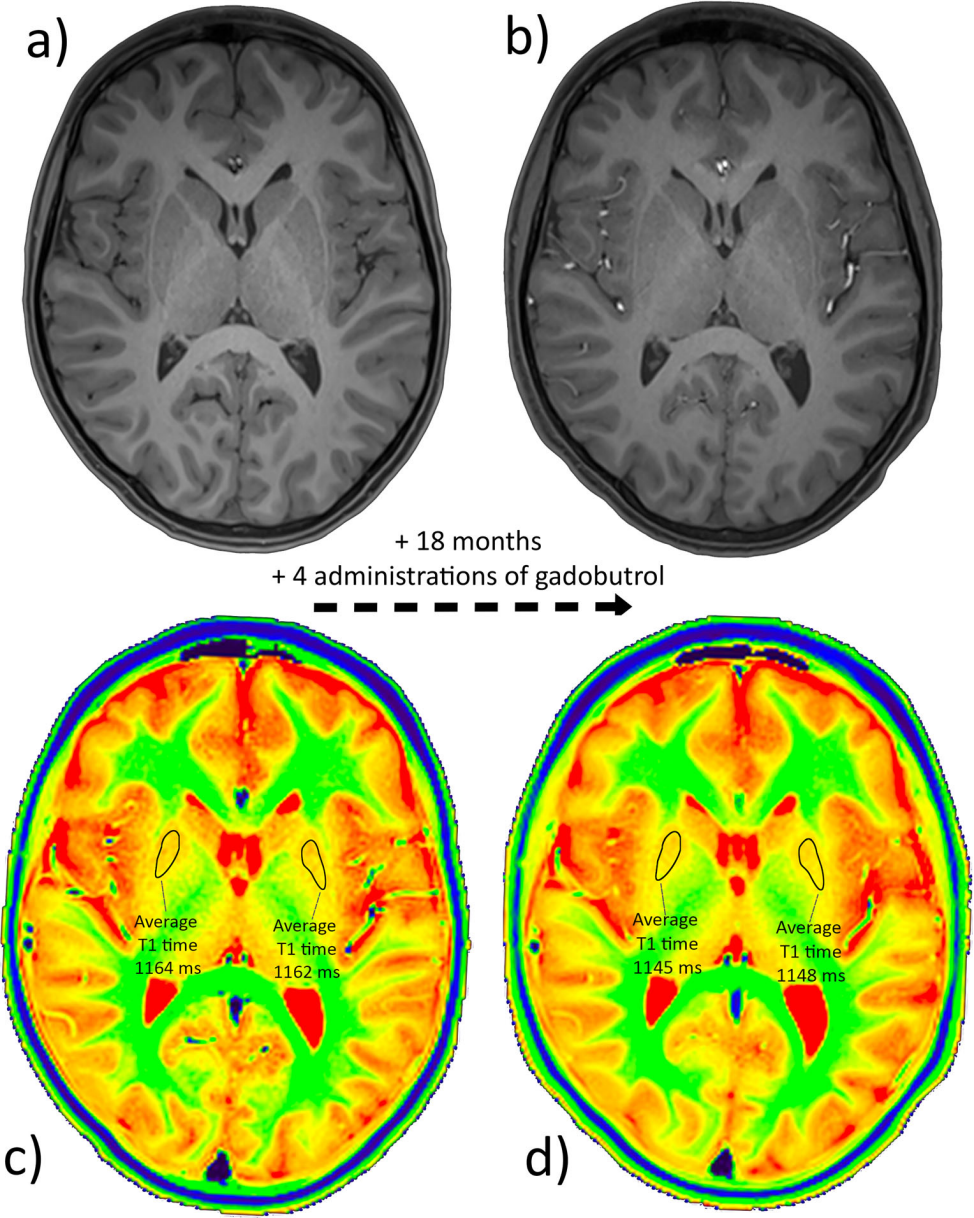
T1-weighted MPRAGE images (**a**, **b**) and T1 maps (**c**, **d**) at the level of the basal ganglia in a 12-year-old girl with status post cerebellar astrocytoma. An interval of 18 months and four administrations of gadobutrol elapsed between the time point (**a**, **c**) and the second time point (**b**, **d**). While the T1-weighted images do not indicate a difference (same windowing), a small decrease of the age-adjusted T1 time from − 6.0 to − 13.5 ms can be measured in the T1 maps of the putamen

On a radiologic workstation (IntelliSpace Portal 10.0, Philips), a freehand tool was used to draw the respective ROI, as previously described in literature [22]. Mean pixel intensity, which equals the T1 relaxation time in milliseconds, was taken (Fig. 2). The following six regions were examined: nucleus caudatus (NC), putamen, GP, thalamus, ND, and frontal white matter (supplemental Figure 1). ROI with posttherapeutic lesions (e.g., resection cavities or gliosis) and disease-related alterations (e.g., demyelination or residual tumor) by employing conventional imaging sequences (T1 weighted, T2 weighted, diffusion weighted) were excluded from further analysis. Each region was measured bilaterally using the mean value, provided there was no pathology. All measurements were performed by two readers (D.G. and S.H.S. with 12 and 8 years of experience in pediatric brain MRI) in independent sessions in each of the two cerebral hemispheres of the patients. Measurements from both readers were employed for interobserver variability, and measurements from D.G. were used for further analysis.

### Statistics

Since the study contained longitudinal data with two measurements for each patient, methods that take the intra-individual correlations into account (mixed models) were used. To determine the expected T1 relaxation time, T1 values from a normative cohort, acquired with the same T1 mapping technique, were employed [22]. The physiological T1 decline during brain development was predicted through a biexponential model. Subsequently, the age-adjusted T1 relaxation time (T1adj) was calculated using the following formula: measured T1 time − expected T1 time given the age. For a pooled analysis of cross-sectional and longitudinal relations, the linear mixed model (LMM) with T1adj as outcome variable and number of gadobutrol administrations, age, and indeterminate effects (as may be caused by disease or therapy) in this non-healthy cohort as covariates was applied.

To validate the results with an alternative analytical method, a generalized additive mixed model (GAMM) was fitted where the T1 relaxation time was modeled as a smooth function of age, a linear function of the number of gadobutrol administrations, and a dummy variable accounting for indeterminate effects of the non-healthy cohort. Those indeterminate effects lead to a fixed shift in T1 time in the gadolinium collective compared with the healthy control collective without correlation to the number of previous gadolinium administration and may be attributable to a variety of causes, but mainly disease or its therapy. The model was fitted using function “gamm” in the R-package “mgcv” with the default parameters (plate regression spline basis) and random intercept for patient. Tests for normal distribution were performed using a quantile-quantile plot and Shapiro-Wilk test. In the case of normal distribution, the values were given as mean and standard deviation, otherwise as median and interquartile range (IQR). In subsequent analyses, the mean values of reader 1 were employed. The interobserver variability between reader 1 and reader 2 was determined through the intraclass correlation coefficient.

All tests on central tendency were two sided. The level of significance was set at 0.05. *p* values were adjusted for multiple comparisons with the Holm-Bonferroni method. Dependence between gender and cohort was determined applying the chi-square test. Statistical analysis was performed using RStudio software (Integrated Development for R. RStudio 1.2.5033, PBC).

## Results

### Cohort

The demographic data and study indications of all 54 patients are presented in Table 1. The age of the patients was not significantly different from that of the normative cohort for calculation of expected T1 relaxation times (median 11.4, *p* = 0.43). A median of 7 (IQR 3–11) doses of gadobutrol had been administered in the history of the patients. Between the first and second examinations, the patients received a median of one dose of gadobutrol (IQR 1–2, min–max 1–9). The median interval between the two timepoints was 238 days (IQR 151–491 days).

**Table 1:** Demographic and clinical data of the study cohort and a previously described normative cohort (median and interquartile range)

	Study cohort	Normative cohort	*p* value
No. of patients	*n* = 52	*n* = 100	
Age (y)	11 (6–15)	11 (7–15)	*p* = 0.43
Gender	Female: *n* = 26	Female: *n* = 54	*p* = 0.6
	Male: *n* = 26	Male: *n* = 46	
Previous gadobutrol doses	7 (3–11)	n.a.	
Intermittent gadobutrol doses	1 (1–2, min–max 1–9)	n.a.	
Clinical indication	Oncologic: *n* = 32	n.a.	
	Multiple sclerosis: *n* = 6		
	Neurofibromatosis: *n* = 4		
	Tuberous sclerosis: *n* = 4		
	Seizures: *n* = 2		
	Inflammation: *n* = 2		
	Focal ischemia: *n* = 1		
	Cerebral fluid spaces: *n =* 1		

### Regression analysis

**Fig. 3:**
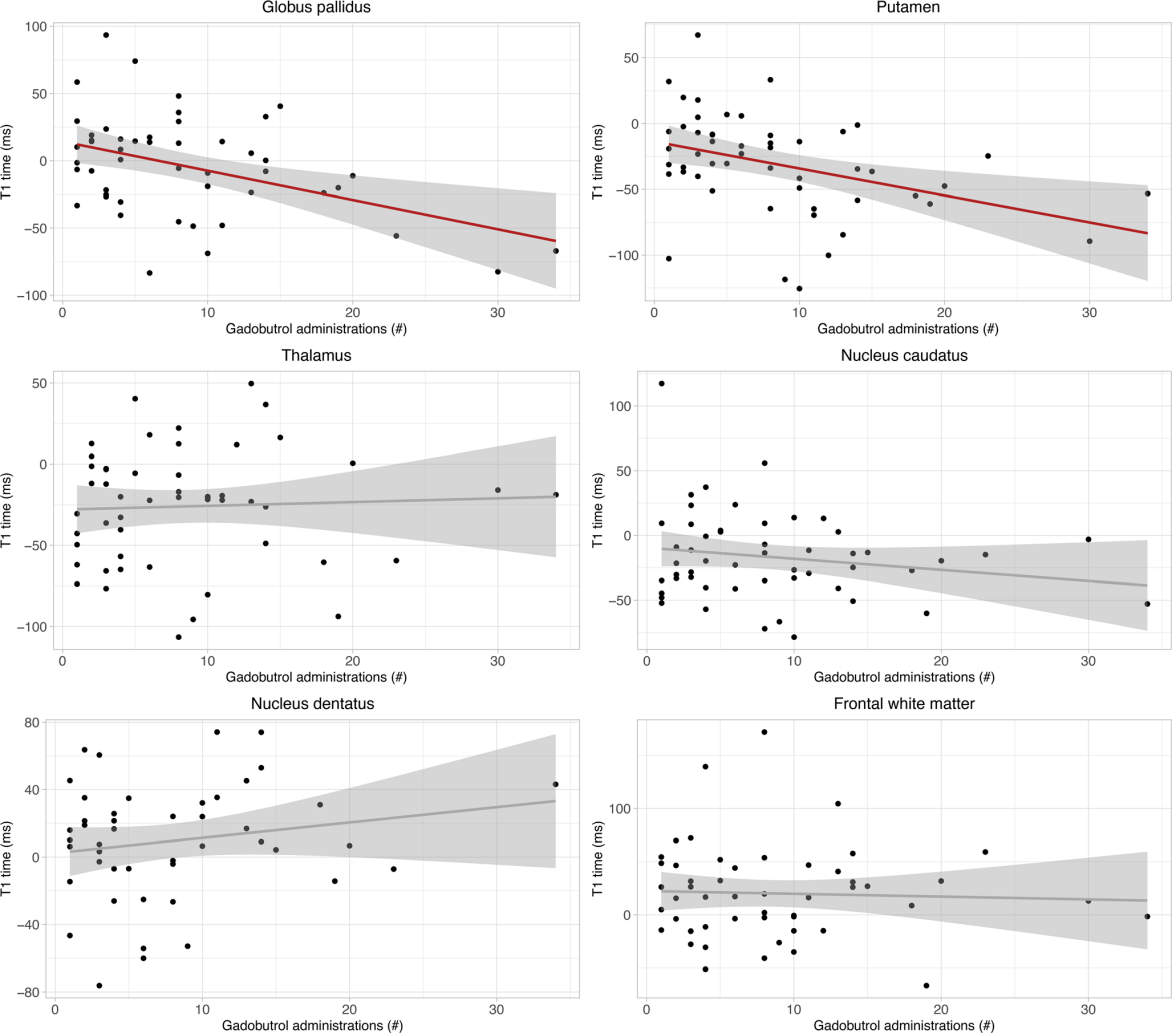
Regression lines (red line) with 95% confidence interval (gray area) of the age-adjusted T1 values of the collective as a function of the number of gadobutrol doses administered so far at timepoint 2. A significant correlation was found in the globus pallidus and putamen (red line)

**Fig. 4:**
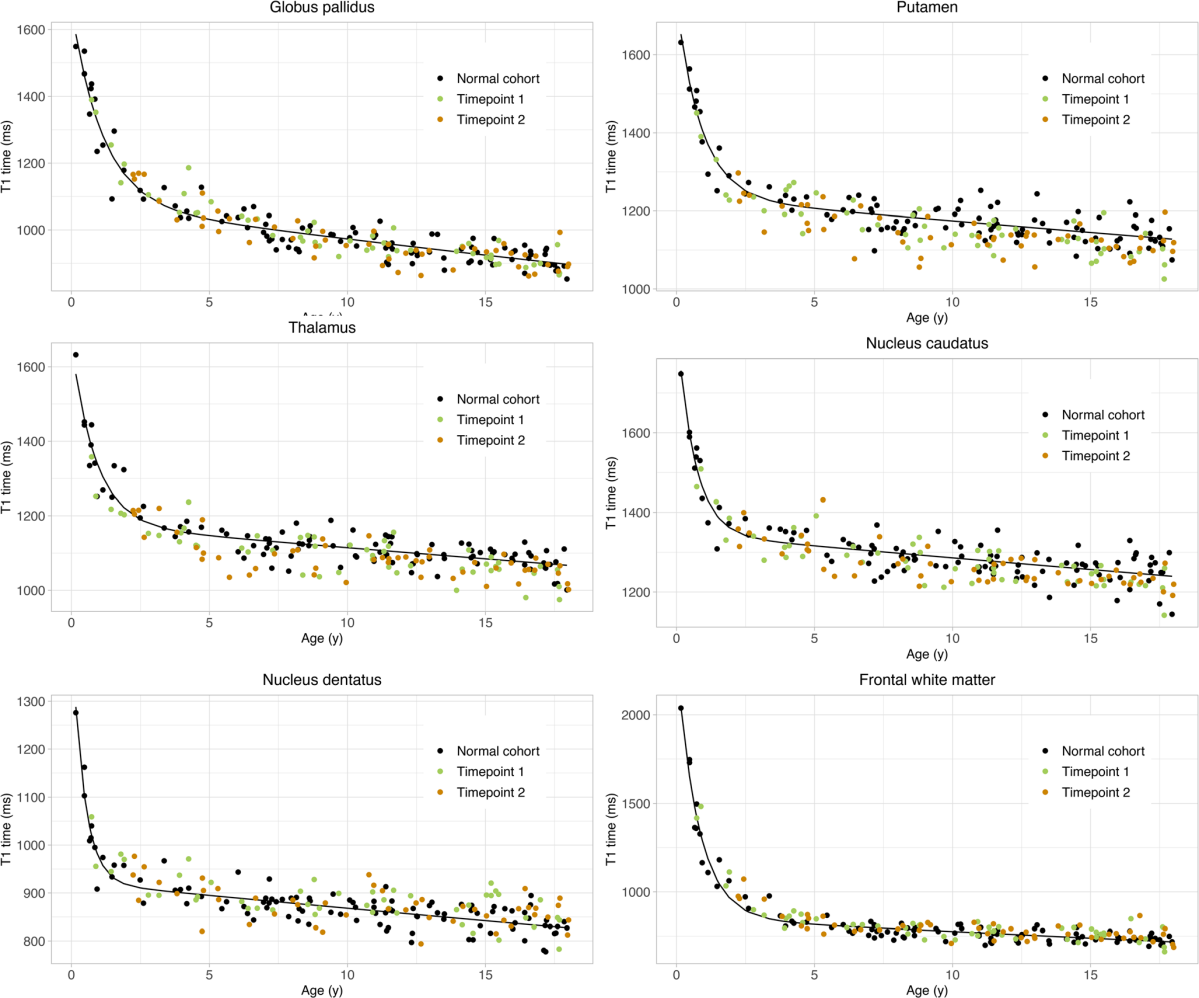
T1 relaxation times in different brain regions in patients with a median of 4 gadobutrol administrations at timepoint 1 (green dots), 6 gadobutrol administrations at time point 2 (orange dots), and contrast agent–naive patients (black dots). Solid line: regression curve of the mean value of the normative cohort

The biexponential model was successful in age-adjusting T1 values with normally distributed residuals in the normal cohort. In linear mixed model analysis, there was a significant decrease of T1adj with the number of gadobutrol administrations in the GP (*p* = .012) and putamen (*p* = .03) (Table 2 and Fig. 3). There was neither a significant decrease in T1adj in the remaining brain regions nor a significant residual effect of age on T1adj in multiple linear regression (supplemental Figure 2). An additional effect on T1 values in the study cohort compared with the normative cohort not caused by age or number of gadobutrol administrations was observed in the GP, putamen, thalamus, and frontal white matter. The results of the linear mixed model were congruent to those obtained with the generalized additive mixed model (supplemental Table 2). The interobserver variability was excellent with an intraclass correlation coefficient greater than 0.96 in each brain region (supplemental Table 3). Figure 4 illustrates the T1 relaxation time raw data for the study and normative cohorts without age adjustments.

**Table 2:** Results for linear mixed model regression after biexponential age adjustment of T1 relaxation for different brain regions. The age-adjusted T1 time is predicted by the number of previous gadobutrol administrations, age, and fixed indeterminate effects (probably caused by disease or therapy) of the study cohort compared to the healthy normal cohort

	Gadobutrol administrations		Patient age		Indeterminate effects	
	*Estimate*	*p value*	*Estimate*	*p value*	*Estimate*	*p value*
Globus pallidus	**− 1.89**	**.012**	− 0.54	0.31	14.33	.15
Thalamus	0.23	> .99	− 0.05	0.93	**− 27.99**	**.006**
Dentate nucleus	0.63	.76	0.24	0.59	8.22	.34
Putamen	**− 1.65**	**.03**	− 0.37	0.49	− 15.21	.15
Caudate nucleus	− 0.76	.76	− 0.55	0.30	− 9.09	.34
Frontal white matter	0.08	> .99	− 0.37	0.59	19.39	.15

Significant effects are displayed in bold-face

## Discussion

This is the first study that demonstrates, in children, a correlation between T1 reduction and former application of a macrocyclic gadolinium-containing contrast agent—in this case, gadobutrol. A significant effect was shown in the GP and putamen. Proving such a correlation in children is challenging because the course of T1 with age is not linear [22], which impedes multiple linear regression analysis. Secondly, there is substantial interindividual variation of brain T1 values. Lastly, irrespective of the number of gadobutrol administrations, there appear to be undetermined effects of the diseases on T1 times. All these challenges are addressed by age adjusting of T1 values employing a normative cohort of the same age range studied with the same methodology as well as by pooled analysis of cross-sectional and longitudinal relations.

In contrast to the use of intensity ratios of T1-weighted images, T1 mapping in adults has been shown to detect more subtle signal alterations, possibly induced by the deposition of gadobutrol. For example, Tedesci et al found a correlation between the number of contrast agent administrations and T1 relaxation time in the ND of 35 adults with multiple sclerosis who had received a mean of 6.3 contrast agent administrations (71% gadobutrol, 8% gadoterate meglumine, another macrocyclic contrast agent, and 20% of a linear contrast agent) [16]. In a multivariate analysis, a borderline effect for macrocyclic contrast media was detectable.

In a cross-sectional study of 46 adult patients, Kang et al determined a significant decrease of T1 in the GP, with an increasing number of preceding gadobutrol administrations [17]. However, radiotherapy was not an exclusion criterion, and 30 patients collectively received radiotherapy to the brain during the course of their disease. In a study group of 160 adults, Saake et al reported a slightly reduced T1 relaxation time in the GP but not in the ND, thalamus, and pons, with a correlation to the number of gadobutrol doses [27].

Another method is quantitative susceptibility mapping. With this technique, Choi et al found increasing susceptibility in GP but not in ND in an adult cohort of 501 patients, with an increasing number of gadobutrol administrations as a potential marker of gadolinium deposition [28].

Histologic evidence as ground truth for deposition of gadobutrol is scarce and prone to bias: Stanescu et al demonstrated gadolinium deposits in various regions of the brain in autopsies of 10 children, only two of whom had received gadobutrol (as well as another contrast agent each) and one of whom had also undergone radiation therapy [9].

The fact that we did not find a significant effect of gadolinium in the dentate nucleus, which was in 2014 the trigger for any further deposition studies, could be explained by several reasons: Besides a possible different kinetics of deposition in children compared to adults, the deposition patterns of linear and macrocyclic contrast agents may differ. Furthermore, the high and variable iron content of this nucleus might impede detection of subtle T1 changes not only interindividually but also intraindividually over time. However, our finding is congruent with a recent T1 mapping study in adults [27].

Both histopathologic studies [9, 29] and the demonstration that gadobutrol can cross a healthy blood-brain barrier via the cerebrospinal fluid [30] suggest that even macrocyclic contrast agents such as gadobutrol, which are considered safer, lead to deposition in brain tissue.

The results of the present study are relevant for more than one reason: First, no non-invasive method has been found to date that verifies brain retention of gadobutrol in children. Second, our findings suggest that the distribution of gadobutrol deposition may differ between adults and children, as significant alterations in T1 are present not only in the GP but also in the putamen. Third, imaging clues are important because histopathologic evidence of deposition is difficult to obtain without confounders such as radiotherapy or administration of other agents in the patient’s history. Taken together, it is reassuring that gadobutrol probably leads to substantially less gadolinium deposition in the brain than linear contrast agents. However, any grade of gadolinium deposition in the brain is of great concern: As a potent calcium antagonist, a variety of possible pathological interactions are conceivable in an organ with omnipresent and vital voltage-gated calcium channels [31]. Yet, it is not clear whether deposition-related long-term effects, if they exist, follow a linear dose-response relationship.

The study has a few limitations beyond its retrospective nature. First, only the status post radiotherapy was an exclusion criterion, not the status post chemotherapy. In addition, according to our results, there are probably other disease- or therapy-related effects in the brain that affect the T1 relaxation time. These limitations largely apply to all MRI gadolinium deposition studies, as these cannot be performed on healthy individuals for ethical reasons. However, to our knowledge, there is no evidence that chemotherapy promotes gadolinium deposition. Another source of error might arise by a hypothetical change in relaxivity, if gadolinium should lose its chelate bond, rendering it less detectable with T1 imaging [32, 33]. Finally, there remains a residual risk; for example, despite questioning the parents and the patient records, a patient might have received a contrast agent other than gadobutrol in an external hospital. However, compared to adults, the probability is low for children, who usually have no concomitant or previous diseases.

In conclusion, the results of the study suggest that based on T1 relaxometry, gadobutrol accumulates in the GP and putamen in children.

## Supplementary Information

ESM 1 (DOCX 1767 kb)

## Abbreviations

GP: Globus pallidus
IQR: Inter-quartile range
ND: Nucleus dentatus
ROI: Region of interest
T1adj: Adjusted T1 relaxation time

## Declarations

### Guarantor

The scientific guarantor of this publication is Daniel Gräfe.

### Statistics and biometry

Maciej Rosolowski kindly provided statistical advice for this manuscript.

### Study subjects or cohorts overlap

Some study subjects or cohorts have been previously reported in Gräfe et al: Quantitative T1 mapping of the normal brain from early infancy to adulthood, Pediatric Radiology volume 51, pages 450–456 (2021).

### Methodology

• retrospective

• case-control study/cross-sectional study

• performed at one institution
