# Supplementary material for: Brain deposition of gadobutrol in children—a cross-sectional and longitudinal MRI T1 mapping study
Source: Eur Radiol. 2022 Dec 15;33(7):4580–8. doi: 10.1007/s00330-022-09297-y (PMC10289941; doi:10.1007/s00330-022-09297-y)

Supplemental table 1: Sequence parameters for conventional MRI sequences for brain imaging

|  | T2 TSE | T2 FLAIR TSE | EPI-DWI | T1 MPRAGE |
| --- | --- | --- | --- | --- |
| Voxel size (mm) | 0.4 x 0.4 x 3.0 | 0.7 x 0.7 x 3.0 | 0.6 x 0.6 x 3.0 | 0.4 x 0.4 x 0.8 |
| TR / TE (ms) | 7870 / 100^a^ | 9000 / 118 | 7100 / 81 | 2000 / 2.57 |
| Flip angle (°) | 150 | 150 | 90 | 8 |
| Inversion time (ms) | n.a. | 2500 | n.a. | 1000 |
| Acquisition mode | 2D | 2D | 2D | 3D |
| Orientation | axial | axial | axial | axial |
| Duration (min) | 3:01 | 3:20 | 1:30 | 5:36 |
| ^a^ 200 ms in infants <12 months  TSE: turbo spin-echo; FLAIR: fluid attenuation inversion recovery; EPI-DWI: echoplanar diffusion weighted imaging; MPRAGE: Magnetization Prepared Rapid Acquisition with Gradient Echoes | | | | |

Supplemental table 2: Results for generalized additive mixed model regression. The effect on T1 time is predicted by a smooth function of age (plate regression spline), the number of previous gadobutrol administrations, and fixed indeterminate effects (probably caused by disease or therapy) of the study cohort compared to the healthy normal cohort.

|  | Gadobutrol administrations | | Indeterminate effects | |
| --- | --- | --- | --- | --- |
|  | *Estimate* | *p-value* | *Estimate* | *p-value* |
| Globus pallidus | **-1.62** | **.036** | 8.13 | .75 |
| Thalamus | 0.35 | .96 | **-31.79** | **0.006** |
| Dentate nucleus | 0.88 | .51 | 2.89 | >.99 |
| Putamen | -1.47 | .10 | **-20.59** | **.035** |
| Caudate nucleus | -0.49 | .96 | -17.30 | .12 |
| Frontal white matter | 1.69 | .28 | 1.99 | >.99 |

Supplemental table 3: Intraclass correlation for different brain regions and two readers (95% confidence interval in brackets).

|  | Interobserver | |
| --- | --- | --- |
|  | *ICC* | *p-value* |
| Globus pallidus | **0.99 (0.99-0.99)** | **<.001** |
| Thalamus | 0.99 (0.99-0.99) | <.001 |
| Dentate nucleus | 0.96 (0.95-0.97) | <.001 |
| Putamen | **0.99 (0.99-0.99)** | **<.001** |
| Caudate nucleus | 0.98 (0.97-0.98) | <.001 |
| White matter | 0.99 (0.99-0.99) | <.001 |

Supplemental figure 1: Regions-of-interest on a T1 map


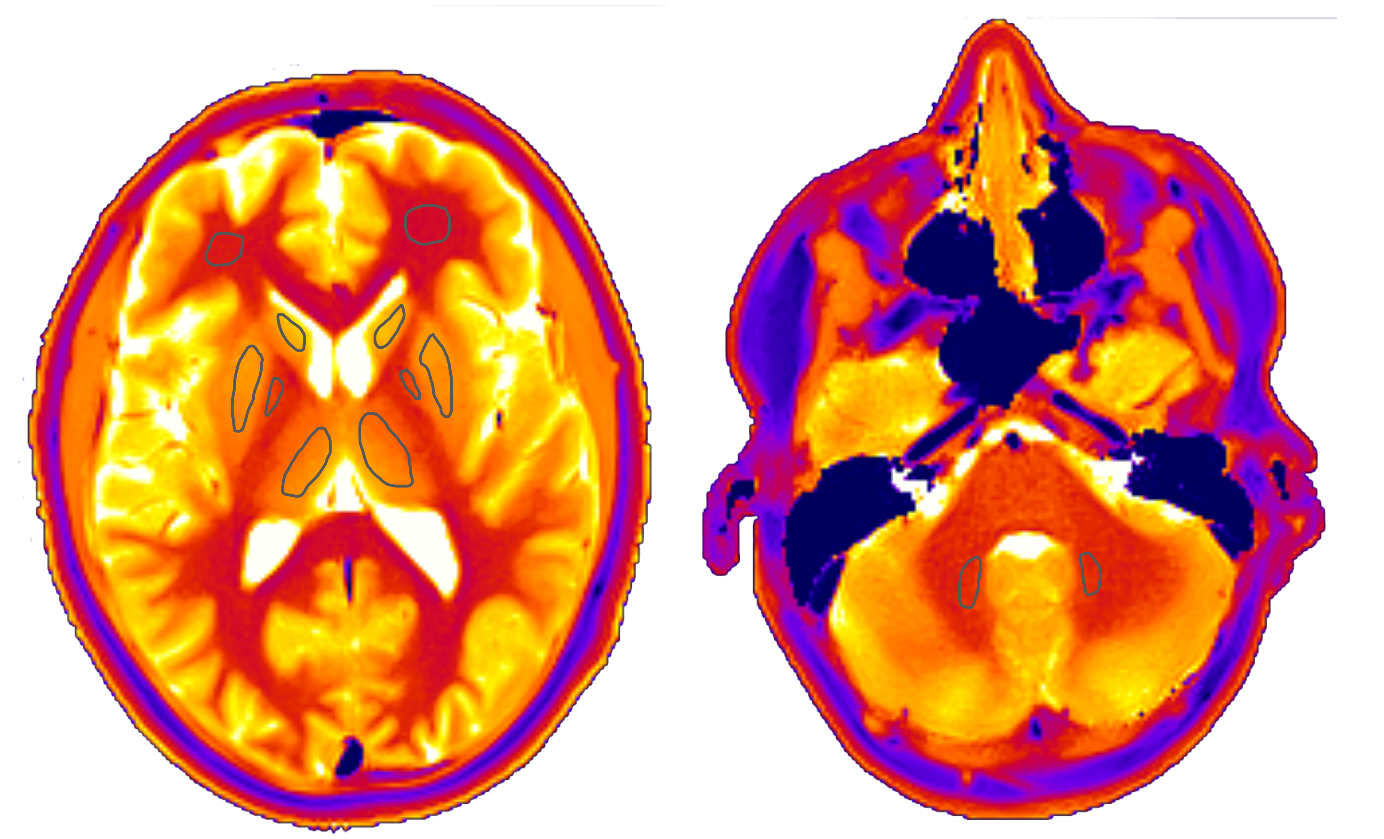


Supplemental figure 2: Distribution of the residuals of the linear mixed model with respect to age of the patient


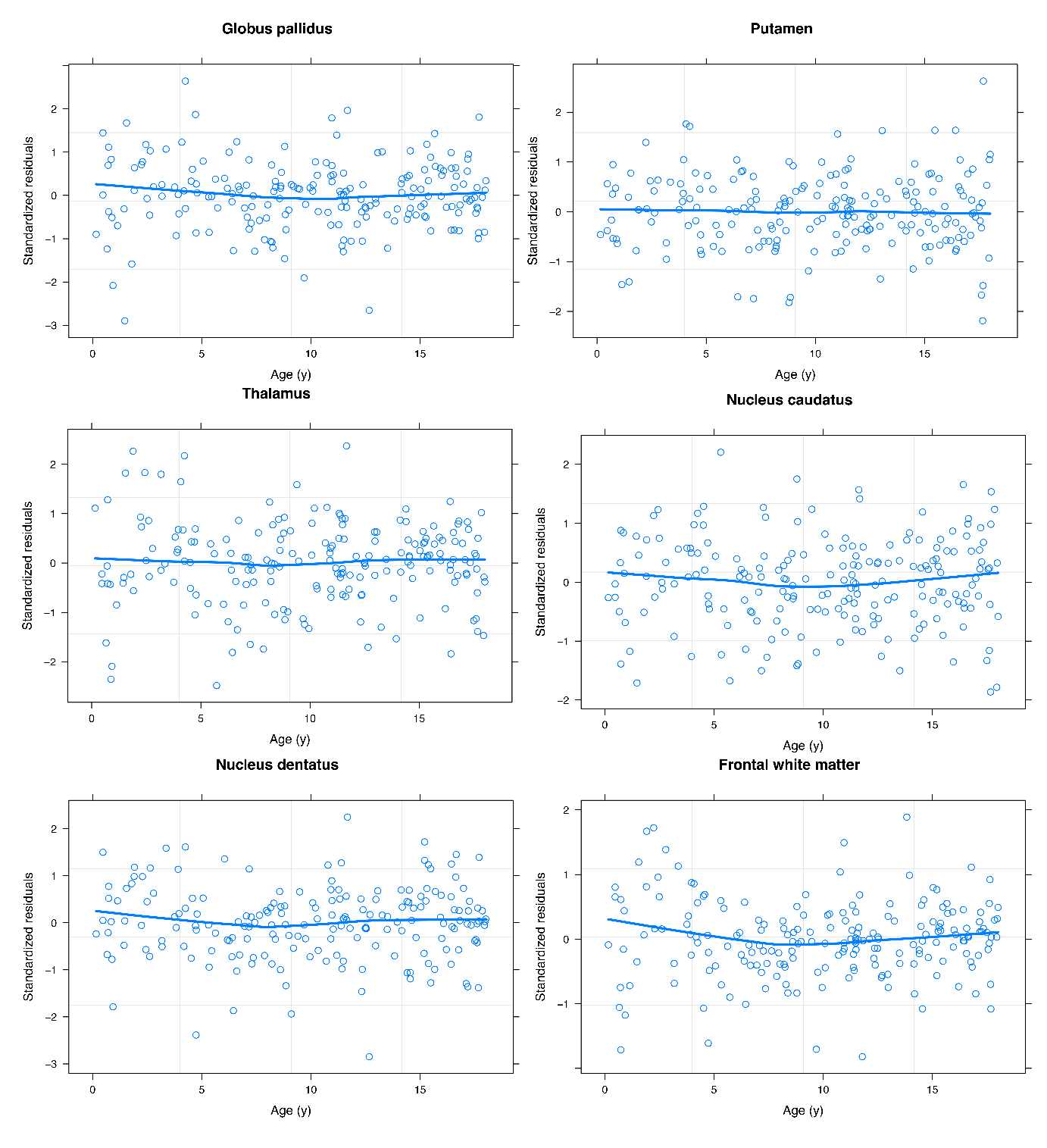

Supplement: Supplementary file 1 — (DOCX 1767 kb) [file 330_2022_9297_MOESM1_ESM.docx]
